# The Impact of Microorganisms on Canine Semen Quality

**DOI:** 10.3390/ani14091267

**Published:** 2024-04-23

**Authors:** Kinga Domrazek, Paweł Konieczny, Marcin Majka, Michał Czopowicz, Piotr Jurka

**Affiliations:** 1 Department of Small Animal Diseases and Clinic, Institute of Veterinary Medicine, Warsaw University of Life Sciences-SGGW, Nowoursynowska 159c, 02-776 Warsaw, Poland; piotr_jurka@sggw.edu.pl; 2 Vet Cell Tech Sp. z o.o., 30-348 Kraków, Poland; pawelk@vetcelltech.com (P.K.); mmajka@cm-uj.krakow.pl (M.M.); 3 Institute of Pediatrics, Faculty of Medicine, Jagiellonian University Medical College, 30-663 Krakow, Poland; 4 Division of Veterinary Epidemiology and Economics, Institute of Veterinary Medicine, Warsaw University of Life Sciences-SGGW, Nowoursynowska 159c, 02-776 Warsaw, Poland; michal_czopowicz@sggw.edu.pl

**Keywords:** mycoplasma, semen quality, CASA-system

## Abstract

**Simple Summary:**

Various microorganisms, including *Mycoplasma* spp., have been reported in canine ejaculate. The impact of these microorganisms on semen quality remains unclear. The aim of this study was to evaluate the prevalence of bacteria and *Mycoplasma* spp. (and various species) in canine semen. Interestingly, 36.5% of the examined dogs tested negative for both aerobic bacteria and mycoplasmas, while 12.7% tested positive for bacterial presence. Additionally, 60.3% of the dogs tested positive for *Mycoplasma* spp. using PCR, with most carrying 1–2 *Mycoplasma* species. We found no significant difference in semen characteristics between *Mycoplasma*-positive and -negative dogs. The detection of *Mycoplasma* was not significantly linked to the presence of bacteria in semen. All the microorganisms identified were classified as saprophytic flora. Some canine ejaculate is sterile. Our findings suggest the existence of undescribed species of canine mycoplasmas, necessitating advanced diagnostic techniques like NGS for their identification.

**Abstract:**

Various microorganisms, including *Mycoplasma* spp., have been reported in canine ejaculate. The impact of these microorganisms on semen quality remains unclear. This study included 63 male intact healthy dogs aged 1–8 years. One dog exhibited azoospermia, indicating a relatively low incidence of this condition. Interestingly, 36.5% of the examined dogs tested negative for both aerobic bacteria and mycoplasmas, while 12.7% tested positive for bacterial presence. Additionally, 60.3% of the dogs tested positive for *Mycoplasma* spp. using PCR, with most carrying 1–2 *Mycoplasma* species. We found no significant difference in semen characteristics between *Mycoplasma*-positive and -negative dogs. The detection of *Mycoplasma* was not significantly linked to the presence of bacteria in semen. All the microorganisms identified were classified as saprophytic flora. Our findings indicate that *Mycoplasma* spp. is common in canine ejaculate. Semen quality parameters were not correlated with the presence of *Mycoplasma* spp. in semen. *Mycoplasma* HRC689 was the most common species. Some dogs exhibited no presence of aerobic bacteria or mycoplasmas in their semen. Our study highlights the common presence of *Mycoplasma* spp. in canine ejaculate. Semen quality shows no correlation with *Mycoplasma* presence. Some canine ejaculate is sterile. Our findings suggest the existence of undescribed species of canine mycoplasmas, necessitating advanced diagnostic techniques like NGS for their identification.

## 1. Introduction

The importance of semen quality in canine reproduction cannot be overstated, as it directly influences the success of breeding programs and the health of the offspring [1]. The evaluation of semen quality encompasses various parameters, including sperm count, motility, morphology, and viability, all of which directly influence the likelihood of successful conception [1]. The contribution of the stud dog constitutes half of the factors that are to be considered when assessing the potential causes of infertility in canine breeding [2]. Due to this fact, the semen quality of the stud dog should be routinely evaluated before mating. Being a carrier of various pathogens is another major factor that needs to be controlled in a dog used for reproduction, as some pathogens may be transmitted via the sexual route to the bitch and lead to reproductive failure [3]. The presence of bacteria in canine ejaculate is a problematic issue in veterinary medicine. Many studies have shown that canine ejaculate is not sterile [4]. It is difficult, however, to distinguish between contamination from the urethra or foreskin and a primary infection of the urogenital tract [4]. Organisms commonly cultured from the semen of healthy male dogs include *Escherichia coli*, *Pasteurella multocida*, beta-hemolytic *Streptococcus*, coagulase-negative *Staphylococcus*, *Staphylococcus pseudintermedius*, *Canicola haemoglobinophilus*, *Klebsiella* spp., and *Pseudomonas* spp. [5,6,7]. Bacterial infections of the urogenital tract can have detrimental effects on canine semen quality, potentially leading to reproductive failures. Some studies describe a negative influence of bacteria belonging to the natural urogenital microbiome, e.g., *E. coli* on fertility [8]. However, except for *Brucella canis*, bacteria appear to be an uncommon cause of compromised fertility in dogs [9]. A negative impact on the seminal quality parameters is likely associated with an increasing number of bacterial species in canine sperm [7]. On the other hand, the bacteria commonly found in semen may play a protective role by inhibiting the growth of pathogenic microorganisms.

The negative influence of bacteria on sperm results from various mechanisms, including direct contact, competition for nutrients, and detritus production [10,11]. Bacterial contamination of ejaculate can lead to decreased spermatozoa motility, increased percentage of dead spermatozoa, and changes in morphology [8,12]. Moreover, after artificial insemination or natural mating, bacteria from ejaculate may induce uterine infections, fertilization failure, embryonic and fetal resorption, abortions, or stillbirths, contributing to decreased litter size and even leading to septicemia in the bitch [13]. One group of bacteria with potential negative impact on semen quality is mycoplasmas.

The data about the occurrence and role of *Mycoplasma* spp. in canine semen are contradictory. Some authors suggest that they have a negative influence on canine fertility [14] and can cause orchitis, epididymitis, and prostatitis [15]. In vitro studies have shown that *Mycoplasma* spp. can be attached to the spermatozoa by interlacing fibrils of variable diameter, which may reduce its motility [16]. Furthermore, Laber and Holtzmann [14] reported a significant increase in the percentage of abnormal spermatozoa and decrease in their motility caused by *M. canis*. *M. maculosum*, and *M. spumans* were described as a cause of 100% of dead forms and 70% of abnormalities in the head, midpiece, and tail of spermatozoa in Bernese Mountain Dogs [12].

Our study aimed to determine the prevalence of aerobic bacteria and mycoplasmas in Polish male dogs and the impact of these microorganism on semen quality.

## 2. Materials and Methods

### 2.1. Study Population and Sampling

This study enrolled adult male intact dogs between 1 and 8 years of age to avoid the potential influence of extreme age on their fertility. These dogs were sourced from kennels affiliated with the Polish Kennel Club (ZKwP, Poland), as well as from shelters for homeless animals. Subsequently, each dog underwent a routine clinical examination to ensure they were free of systemic diseases, and serum testosterone, estradiol, and total thyroxin concentrations were measured to eliminate the potential influence of endocrine disorders on semen quality.

All medical procedures were performed as a part of routine veterinary examination on the owners’ request and thus, according to the European directive EU/2010/63 and Polish legal regulations, the approval of Ethical Committee for the described procedures was not required, as they could be qualified as nonexperimental clinical veterinary practices excluded from the directive (Act of 15 January 2015 on the protection of animals used for scientific or educational purposes).

Eventually, 63 clinically healthy male dogs with the aforementioned hormones within the reference intervals were enrolled in study. Semen was collected in the sterile containers by digital manipulation, and the sperm-rich fraction of the ejaculate was analyzed according to standards [1]. From each semen sample, the swab was collected and sent to the commercial laboratory (Vetlab, Warsaw, Poland) for the routine bacteriological examination. Additionally, three cotton swabs were taken from each semen sample and air-dried. One of these swabs was sent to the same commercial veterinary laboratory (Vetlab, Poland) for PCR for *Mycoplasma* spp., canine herpesvirus type 1 (CHV-1), and *Chlamydia* spp., while the remaining two were kept at −80 °C until the results of PCR had been obtained. Based on the PCR results, dogs were categorized into the *Mycoplasma*-positive or *Mycoplasma*-negative group. The samples from *Mycoplasma* spp.-positive dogs were further analyzed to identify the exact *Mycoplasma* species. No samples were positive for CHV-1 or *Chlamydia* spp., as described elsewhere [17].

### 2.2. Hormone Measurements

After clotting, blood samples were centrifuged at 2057× *g* for 5 min, and serum was harvested. Hormones were quantified using the competitive enzyme immunoassay competition method with final fluorescent detection (ELFA) (MINI, VIDAS, bioMérieux, Marcy l’Etoile, France) in accordance with the manufacturers’ manuals. Reference intervals were defined as follows: testosterone ≥ 1 ng/mL [18], estradiol < 115 pg/mL [19], and total thyroxin within the range of 10–50 nmol/L [20].

### 2.3. PCR Analyses

To increase the efficiency of the reactions, two swabs were used to carry out the PCR reaction. DNA isolation was performed using the Swab-Extract DNA Purification Kit (Eurx, Gdańsk, Poland), following the manufacturer’s guidelines. PCR reactions were performed using recently published primers specific to various *Mycoplasma* species [21], along with Taq PCR Master Mix (2x) (Eurx, Gdańsk, Poland). Protocols for PCR were adapted from standard procedures described previously [22,23]. Subsequently, the PCR products were analyzed via electrophoresis in a 2% agarose gel, and the approximate lengths of the amplicons were determined using a molecular-weight size marker (100 bp DNA ladder) as a reference.

### 2.4. Bacteriological Examination

The semen samples were collected for bacteriological tests using transport agar medium swabs and promptly send to the commercial laboratory (Vetlab, Poland). The samples were cultured on the following microbiological media: Columbia agar with 5% ovine blood, MacConkey agar, Columbia CNA agar with 5% ovine blood, and chocolate agar. Incubation conditions included maintaining a temperature of 35–37 °C for 48 h in an oxygen atmosphere (Columbia agar with 5% ovine blood, MacConkey agar, and Columbia CNA agar with 5% ovine blood) or an atmosphere with an elevated concentration of CO_2_ (Chocolate Agar), facilitated by a CO_2_ atmosphere generator (Gen Compact, bioMérieux, Marcy l’Etoile, France). The bacterial growth was reviewed 24 and 48 h post-incubation. Subsequently, the obtained bacterial colonies underwent analysis in the MALDI TOF Biotyper Sirius IV (Billerica, MA, USA).

### 2.5. Semen Quality Evaluation

#### 2.5.1. Macroscopic Evaluation

The volume of the sperm-rich fraction was measured by using calibrated pipettes, and the color of the semen was visually assessed. Cloudy or milky opacity were considered normal, following guidelines outlined by Root Kustritz [24]. The pH value was determined in each semen sample by dipping litmus strips.

#### 2.5.2. Morphology Evaluation

The morphology of spermatozoa was evaluated by preparing smears from the second fraction, which were then air-dried and immersed in the sperm stain (Microptic, Barcelona, Spain) for 5 min. Then, the samples were examined in the light microscope (ECLIPSE E 200, Nikon, Tokyo, Japan) at 100-fold magnification. At least 200 spermatozoa were reviewed and categorized according to the criteria established by Freshman [1] into normal spermatozoa and spermatozoa exhibiting defects of the head, midpiece, or tail. Semen samples with more than 70% of spermatozoa of normal morphology were classified as physiological [1].

#### 2.5.3. Viability Evaluation

The conventional microscopic assessment of the proportion of viable (with intact cell membrane) and dead (with compromised cell membrane) spermatozoa was conducted using nigrosine–eosin stain according to established protocols [25]. A warm mixture of the stain and semen (comprising 3 μL of eosin, 3 μL of nigrosine, and 3 μL of semen) was smeared on a heated glass slide [26] and air-dried. Then, the samples were examined under the light microscope (ECLIPSE E 200, Nikon, Tokyo, Japan) at 100-fold magnification. Each assessment involved the evaluation of at least 200 cells, with the results presented as the percentage of viable and dead spermatozoa.

### 2.6. Computer-Assisted Sperm Analysis (CASA)

The computer-assisted sperm analysis (CASA) was conducted using the sperm class analyzer (SCA version 6.5.0.67, Microptic, Barcelona, Spain) in conjunction with the light microscope (ECLIPSE E 200, Nikon, Tokyo, Japan) and camera (Basler, Ahrensburg, Germany). The thermostable table of the analyzer was heated to a temperature of 37 °C according to the established protocols [27]. The sperm-rich semen fraction was diluted in the proportion 1:1–1:5 with phosphate-buffered saline (PBS, Sigma Aldrich, Saint Louis, MI, USA) and incubated for 5 min at 37 °C prior to evaluation. Analysis was performed using a 20-micron GoldCyto 4-chamber slide (Goldcyto Biotech corp., Shanghai, China), using the manufacturer settings for dogs, as follows: VLC Rapid 165 μm/s, Lin Rapid 55%, and the average head area 20 μm^2^.

In each analysis, a minimum of 500 spermatozoa were counted and examined for the following characteristics: concentration, motility, mucus penetration, and round cell count. Additionally, spermatozoa were categorized into subpopulations based on their movement characteristics, including velocity (fast (RAPID), moderate (MEDIUM), slow (SLOW)), direction (progressive, moderately progressive, nonprogressive), and the percentage of spherical tracks. A total spermatozoa count exceeding 200 × 10^6^ and the percentage of motile spermatozoa exceeding 70% was considered normal [24]. To ensure the reliability of the results, all semen samples were microscopically evaluated by the same highly qualified staff member.

### 2.7. Statistical Methods

Categorical variables were presented as counts of groups and percentages from this study population and compared between groups using the likelihood ratio G test or Fisher exact test (if any expected cell count in the contingency table was <5). The 95% confidence interval (CI 95%) for proportions was calculated using the Wilson score method [28]. Numerical variables were tested for normality of distribution through the inspection of normal probability Q-Q plots and using the Shapiro–Wilk W test. As normality assumption was violated in most cases, the numerical variables were expressed as the median, interquartile range (IQR), and range and compared between groups using the Mann–Whitney U test. Their correlations were tested using Spearman’s rank correlation coefficient (R_s_). All statistical tests were 2-tailed, and the significance level (α) was set at 0.05. Statistical analysis was performed using TIBCO Statistica 13.3 (TIBCO Software Inc., Palo Alto, CA, USA).

## 3. Results

### 3.1. Study Population

This study included sixty-three male, intact, clinically healthy dogs aged from 1 to 8 years with a median (IQR) of 3.0 (1.5–4.5) years; twenty-three dogs (36.5%) were 1 year old, eight dogs (12.7%) were 2 years old, nine dogs (14.3%) were 3 years old, nine dogs (14.3%) were 4 years old, five dogs (7.9%) were 5 years old, two dogs (3.2%) were 6 years old, three dogs (4.8%) were 7 years old, and four dogs (6.3%) were 8 years old. Three dogs were crossbreeds (4.8%), and the remaining sixty dogs belonged to forty-seven breeds, among which Border Collie was represented by six dogs and springer spaniel and English Mastiff were represented by three dogs (Table S1). Body weight ranged from 3 to 120 kg, with a median (IQR) of 24 (15–31) kg.

Testosterone and estradiol concentrations were within the reference interval in all dogs. Total thyroxin concentration was lowered in twelve dogs (only in two dogs had <10 nmol/L) and slightly elevated in one dog (56.5 nmol/L).

### 3.2. Semen Characteristics

Only 1/63 dogs (1.6%; CI 95%: 0.3–8.5%) had azoospermia, and 5/63 dogs (7.9%; CI 95%: 3.4–17.3%) had oligospermia (<200 × 10^6^ sperms). Abnormal spermatozoa morphology (≤70% of normal spermatozoa in semen) was found in 3/62 dogs (4.8%, CI 95%: 1.7–13.3%). Details of semen characteristics are presented in Table 1. Semen volume was significantly positively correlated with the body weight of dogs (R_s_ = 0.37, *p* = 0.003), while round cell count and the proportion of normal spermatozoa was significantly correlated with the age of dogs, the former positively (R_s_ = 0.34, *p* = 0.006) and the latter negatively (R_s_ = −0.35, *p* = 0.005)

### 3.3. Bacteriological and PCR Findings

In 8/63 dogs (12.7%, CI 95%: 6.6–23.1%), the following aerobic bacteria were cultured from the semen: *Staphylococcus pseudintermedius* in 3 dogs, *Streptococcus canis* in 2 dogs, followed by *Staphylococcus vitulinus*, *E. coli*, and *Pseudomonas* sp. in 1 dog each. *Mycoplasma* spp. was detected using PCR in 38/63 dogs (60.3%; CI 95%: 48.0–71.5%). In 10/38 *Mycoplasma*-positive dogs (26.3%), the *Mycoplasma* species could not be determined using routine PCR primers. In the remaining 28 dogs, 54 *Mycoplasma* strains belonging to twelve species were identified (Table 2)—one species in 11/28 dogs (39.3%), two species in 10 dogs (35.7%), three species in 5 dogs (17.9%), and four species in 2 dogs (7.1%). Except for three dogs with *M. canis*, two dogs with *M. haemocanis*, and two dogs with M. HRC689, all other *Mycoplasma*-positive dogs had unique combinations of various *Mycoplasma* species (Table 3). The detection of *Mycoplasma* was not significantly associated with the presence of bacteria in the semen (*p* = 0.461).

### 3.4. Relationship between the Presence of Mycoplasma and Semen Characteristics

There was no significant difference in demographic and hormonal characteristics between *Mycoplasma*-positive and *Mycoplasma*-negative dogs (Table S2). The presence of *Mycoplasma* in the semen did not prove to be associated with any significant changes in the semen characteristics (Table S3).

## 4. Discussion

To the best of our knowledge, this is the first study in which PCR testing for all known canine mycoplasmas has been performed on canine semen material. Lechner et al. tried to detect only six of them [6]. Schafer-Somi et al. evaluated nine species of canine *Mycoplasma* in semen by culturing them [29]. Tamiozzo performed gene sequencing and detected only two species of these bacteria [12]. Currently, in routine veterinary practice, the gold standard for mycoplasma diagnosis is PCR testing, so our study focused on this method. Commercially available laboratories detect only *Mycoplasma* spp. without species identification of this bacteria. This leads to a lack of available statistics on the prevalence of exact species. Moreover, the knowledge regarding which species are pathogenic and which are not makes the obtained results difficult to interpret.

Studies suggest that *Mycoplasma* spp. may be present in the reproductive tract of dogs at varying rates, with estimates ranging from 30% to 89% [15,30]. In our study, the prevalence of *Mycoplasma* spp. in canine ejaculate was 60%. Schafer-Somi et al. detected these bacteria in 55% of samples, including 35% of samples of good-quality ones [29]. The prevalence seems to be similar, but the methodology is significantly different. Interestingly, the most frequently detected species of *Mycoplasma* was in our study—*Mycoplasma* HRC689. The presence of this *Mycoplasma* species in canine semen has not been investigated so far. *M. cynos* [6] or *M. canis* [29] appear to be the most common *Mycoplasma* species in ejaculate. In our study, these two species were detected in 5.3% and 18.4% of tested dogs, respectively. Our data did not show any significant correlation between various species of this bacteria and semen quality, while Tamiozzo suggested that *M. spumans* and *M. maculosum* negatively affected male dogs’ fertility [12]. Also, in another study, *Mycoplasma* was detected in a significantly higher percentage of poor-quality ejaculate samples compared to ejaculate samples of good quality [29].

Our results show that the detection of *Mycoplasma* spp. was not significantly associated with the presence of bacteria in the semen. This phenomenon could be caused by several factors. First, too small of a sample size could have undermined the statistical power required to detect meaningful differences. Consequently, even if a genuine association had existed, it may have remained undetected. Secondly, high variability in the methodologies employed for *Mycoplasma* and bacterial detection, encompassing diverse approaches, such as culture-based methods and molecular assays like polymerase chain reaction (PCR), could introduce disparities in their diagnostic sensitivity or specificity. In our opinion, using NGS technology could shed more light on these aspects. More research in this field is needed. The last explanation of this phenomenon could be the coincidental presence of those microorganisms. The co-occurrence of *Mycoplasma* and bacterial species in semen may be incidental rather than reflective of a direct causal relationship. Shared transmission routes, such as sexual activity, or similar ecological niches within the reproductive tract, could facilitate coincidental cohabitation without necessitating an intrinsic association.

Among the aerobic bacteria isolated from the semen samples in our study, various species were identified, including *Staphylococcus* spp., *Streptococcus* spp., and *E. coli.* These results are consistent with data reported in the available literature [6]. Our study also showed that not all ejaculate contained aerobic bacteria. In only 12.7% of samples, aerobic bacteria were cultured. This result is contradictory to other studies, which suggested that canine semen is not sterile [5,6,7]. On the other hand, data evaluated by another author suggested that the source of bacteria could be an environment, bacteria on the urethra [31], or a lack of proper hygiene of the person who collect the samples. Regardless of the quality of semen, bacterial growth is observed in various fractions of dog semen. However, higher concentrations are typically found in the first fraction, which is primarily attributable to the presence of bacteria originating from the urethra [4]. Dogs included in our study did not show any signs of urinary tract infection. The samples were collected with clean gloves in sterile containers. The samples for bacteriology were collected according to rules that are used, e.g., during urine collection, which means that the middle stream of semen was collected for bacteriology [32]. On the one hand, there are studies that describe the presence of bacteria as physiological [33], and on the other hand, some others consider bacteriospermia as pathology [7,8]. The number of bacteria and the immune status of the organism matter. Typically, the detection of over 10,000 colony-forming units of aerobic bacteria per milliliter of semen indicates an infection of the genital tract [34]. The infection is generally correlated with presence of inflammatory cells [35]. In our study there were no significant differences in round cell concentration in semen and bacterial or mycoplasmal contamination. Similar results have been obtained in the analysis of the cytology of seminal fluids performed by Kustritz et al. [36].

The predominant components of the physiological microflora in female dogs typically comprise β-hemolytic *Streptococcus* spp., *Staphylococcus* spp., *E. coli*, *Enterococcus faecalis*, *Pasteurella multocida*, *Proteus* spp., *Bacillus* spp., *Corynebacterium* spp., *Klebsiella pneumoniae*, *Actinomyces* spp., and *Neisseria* spp. Additionally, certain authors propose the presence of *Lactobacillus* spp., *Mycoplasma* spp., and *Ureaplasma* spp. [37,38]. Our bacteriology results obtained from fertile dogs indicate that the saprophytic flora of the male reproductive tract is similar. This suggests that prophylactic antibiotic therapy after positive bacteriology results in dogs with normal parameters describing semen is not justified because similar microorganisms inhabit the body of the bitch, and there is no risk of infecting her.

The findings of this study have practical implications for veterinary practice and breeding programs. By identifying the microbial flora present in canine ejaculate and its influence on fertility parameters, this study contributes to the development of targeted screening and management protocols to improve breeding success rates and reproductive outcomes in dogs. In the current veterinary practice, the carriers of *Mycoplasma* spp. are mainly treated using doxycycline [39]. Our findings show that not every carrier of this bacteria should be treated. This result appears to be extremely important, as it will help reduce the use of antibiotic therapy in veterinary medicine. The overuse and misuse of antibiotics contribute to the development of antibiotic-resistant bacteria [40]. When antibiotics are used too frequently or inappropriately, bacteria can evolve and become resistant, making infections more difficult to treat [40]. In addition, antibiotics not only target harmful bacteria but can also affect the beneficial bacteria in the body, disrupting the natural balance of the microbiome. This disruption can lead to various health issues, including digestive problems and increased susceptibility to infections [41]. Due to this fact, it is highly recommended to evaluate semen quality, including the presence of inflammatory cells after obtaining bacteriology or PCR results from ejaculate. The decision on treatment should be made after careful consideration of all the factors.

Our study also has some limitations. While this study provides valuable insights into the prevalence and impact of aerobic bacteria and mycoplasmas in Polish male dogs, the findings may not be directly generalizable to other canine populations in different geographic regions. Factors such as breed diversity, environmental conditions, and management practices could influence the microbial composition of semen. This study focuses specifically on male dogs from Poland, which may limit the applicability of the findings to dogs from other countries or regions with different environmental conditions and management practices. Including participants from multiple geographic locations could enhance the external validity of this study. This study provides a cross-sectional snapshot of semen quality and microbial presence in male dogs at a specific point in time. Longitudinal data tracking changes in semen quality and microbial composition over time could provide deeper insights into the dynamic nature of these factors. The final limitation of our study is that the diagnostic methods we used are qualitative, not quantitative. It is possible that the quantity of bacteria has a greater impact on semen quality than the species themselves. More research in this area is needed. Our methodology, which contains PCR reactions and bacteriology culturing, is not cutting-edge technology, but it is available to both scientists and veterinarians. In the future, we want to expand our research to include the use of technology next-generation sequencing (NGS) [42]. Another limitation correlated with methodology is using basic diagnostic tools like CASA-system and microscopy evaluation of the morphology and viability of sperms. Our methodology, which includes diagnostic tools such as the CASA system, microscopic evaluation of sperm morphology and viability, PCR reactions, and bacteriological culture, are widely available to both researchers and clinical veterinarians. The use of these testing methods provides reproductive veterinarians, including practitioners and clinicians, with accessible tools for diagnosing infertility in their routine practice. Therefore, we also chose to use a simple eosin–nigrosine test as a surrogate for assessing functional membrane integrity. This test distinguishes between damaged and intact cell membranes, with damaged membranes staining pink while intact membranes remain unstained. While more sophisticated techniques, such as the hypoosmotic edema test (HOS), are typically available primarily in research settings, we anticipate incorporating them into our future research, similarly to next-generation sequencing (NGS) technology. Using these basic but effective tests, we aim to improve the diagnostic process for reproductive veterinarians and facilitate the timely and accurate identification of semen quality problems in dogs.

This study encountered challenges in identifying specific *Mycoplasma* species in some cases, with 10 out of 38 *Mycoplasma*-positive dogs having unidentified species. This limitation could affect the accuracy of the associations between *Mycoplasma* species and semen quality parameters. The presence of a positive result for *Mycoplasma* spp. and the absence of a positive result in PCR reactions for known species may suggest that these dogs were carriers of another species. The ideal solution to this situation would be to sequence genes from samples obtained from these dogs. This will be the direction of our further research.

The significance of this study lies in its contribution to understanding the factors affecting reproductive health in dogs. This research addresses a critical gap in current knowledge by investigating the prevalence and impact of aerobic bacteria and mycoplasmas on semen quality, which is a crucial aspect of canine fertility and breeding success. By identifying and characterizing the microbial flora present in canine ejaculate, this study sheds light on potential sources of contamination and infection that may compromise semen quality. Understanding the microbial composition of semen and its influence on fertility parameters is essential for developing effective strategies to optimize reproductive outcomes in dogs. Furthermore, the study findings may have practical implications for veterinary practice and breeding programs. By elucidating the role of aerobic bacteria and mycoplasmas in semen quality, veterinarians and breeders can implement targeted screening and management protocols to minimize the risk of reproductive tract infections and improve breeding success rates.

## 5. Conclusions

In conclusion, in canine ejaculate, *Mycoplasma* spp. is common in dogs that have not been used for reproduction. The semen quality parameters are not related to the general presence of *Mycoplasma* spp. The most common species is *Mycoplasma* HRC689. There are dogs in whose semen neither aerobic bacteria nor mycoplasmas are present, which indicates that in some cases, the semen could be sterile. It is likely, however, that there are yet undescribed species of canine mycoplasmas that cannot be detected using conventional diagnostic tools. Therefore, further investigations employing advanced techniques, such as NGS, are imperative to unveil these elusive pathogens.

## Figures and Tables

**Table 1:** General characteristics of the semen of study dogs.

Semen Characteristics ^a^	Median	Interquartile Range (Range)
General semen characteristic		
Semen volume [mL]	2.5	1.5–3.5 (0.4–5.0)
pH	6.0	6.0–6.5 (3.0–7.5)
Sperm concentration [×10^6^/^mL^]	365.7	204.3–596.6 (42.2–1649.3)
Total sperm number [×10^6^]	671.2	398.9–1401.7 (113.4–3298.7)
Oval cell count [×10^6^/^mL^]	1.0	0.4–3.0 (0–17.7)
Spermatozoa morphology		
Normal spermatozoa [%]	92.5	88.0–95.0 (58.5–99.0)
Head abnormalities [%]	1.8	1.0–4.0 (0–18.0)
Midpiece abnormalities [%]	2.0	1.5–3.5 (0–20.0)
Tail abnormalities [%]	2.8	1.0–5.0 (0–29.5)
Abnormal spermatozoa [%]	7.5	5.0–12.0 (1.0–41.5)
Spermatozoa motility		
Total motility [%]	93.4	87.7–96.4 (34.4–99.8)
Progressive motility [%]	31.7	21.2–39.4 (0.7–55.9)
Medium-progressive motility [%]	32.1	26.5–45.9 (4.6–78.7)
Non-progressive motility [%]	23.6	17.8–29.5 (5.1–49.5)
Spherical tracks [%]	38.3	31.4–49.7 (1.2–83.7)
Rapid motility [%]	57.4	45.6–68.4 (3.7–94.2)
Medium motility [%]	24.7	17.6–31.5 (5.1–53.4)
Slow motility [%]	7.0	4.4–10.9 (0.5–38.8)
Mucus penetration [%]	31.2	20.7–41.1 (2.4–64.9)
Viability [%]	91.8	87.0–95.0 (40.0–98.5)

^a^ spermatozoa characteristics for 62 dogs that had spermatozoa in semen.

**Table 2:** Species of *Mycoplasma* spp. detected in canine semen.

Mycoplasma Species	Number of Dogs	Prevalence (CI 95%) [%]
M. HRC689	13/38	34.2 (21.2–50.1)
*M. canis*	7/38	18.4 (9.2–33.4)
*M. haemocanis*	6/38	15.8 (7.4–30.4)
*M. arginini*	5/38	13.2 (5.8–27.3)
*M. VJC365*	4/38	10.5 (4.2–24.1)
*M. molare*	3/38	7.9 (2.7–20.8)
*M. maculosum*	3/38	7.9 (2.7–20.8)
*M. feliminutum*	3/38	7.9 (2.7–20.8)
*M. edwardii*	3/38	7.9 (2.7–20.8)
*M. opalescens*	3/38	7.9 (2.7–20.8)
*M. cynos*	3/38	5.3 (1.5–17.3)
*M. bovigenitalium*	3/38	5.3 (1.5–17.3)
Unidentified	10/38	26.3 (15.0–42.0)

**Table 3:** The combinations of *Mycoplasma* species detected in canine semen.

Mycoplasma spp.	Number of Dogs
1 *Mycoplasma* species	
*M. canis*	3
*M. haemocanis*	2
M. HRC689	2
*M. arginini*	1
*M. edwardii*	1
*M. molare*	1
M. VJC 358	1
10 Combinations of 2 *Mycoplasma* species	
M. HRC689 and *M. canis*	1
M. HRC689 and *M. cynos*	1
M. HRC689 and *M. edwardii*	1
M. HRC689 and *M. arginini*	1
M. HRC689 and *M. feliminutum*	1
M. HRC689 and *M. bovigenitalium*	1
M. HRC689 and *M. haemocanis*	1
M. VJC358 and *M. haemocanis*	1
M. VJC358 and *M. feliminutum*	1
*M. arginini* and *M. molare*	1
5 Combinations of 3 *Mycoplasma* species	
M. HRC689 and *M. cynos* and *M. arginini*	1
M. HRC689 and *M. maculosum*	1
M. HRC689 and *M. canis* and *M. molare*	1
M. VJC358 and *M. feliminutum* and *M. opalescens*	1
*M. haemocanis* and *M. maculosum* and *M. opalescens*	1
2 Combinations of 4 *Mycoplasma* species	
M. HRC689 and *M. canis* and *M. maculosum* and *M. bovigenitalium*	1
*M. canis* and *M. edwardii* and *M. opalescens* and *M. haemocanis*	1

## Data Availability

No new data were created or analyzed in this study. Data sharing is not applicable to this article.

## Supplementary Materials

The following supporting information can be downloaded at: https://www.mdpi.com/article/10.3390/ani14091267/s1, Table S1. Breeds of dogs of the study population; Table S2. Demographic and hormonal characteristics of dogs from Mycoplasma-positive and Mycoplasma-negative group; Table S3. Influence of the presence of *Mycoplasma* in the semen on semen characteristics.

## References

[1] Freshman J.L. (2002). Semen Collection and Evaluation. Clin. Tech. Small Anim. Pract..

[2] Kolster K.A. (2018). Evaluation of Canine Sperm and Management of Semen Disorders. Vet. Clin. N. Am. Small Anim. Pract..

[3] England G.C.W., Burgess C.M., Freeman S.L. (2012). Perturbed Sperm–Epithelial Interaction in Bitches with Mating-Induced Endometritis. Vet. J..

[4] Luño V., González N., Martínez F., Revert A., Morrell J.M., Gil L. (2020). Colloid Centrifugation Reduces Bacterial Load in Chilled Dog Semen. Anim. Reprod. Sci..

[5] Kustritz M.V.R. (2006). Collection of Tissue and Culture Samples from the Canine Reproductive Tract. Theriogenology.

[6] Lechner D., Aurich J., Spergser J., Aurich C. (2023). Double Semen Collection at a 1-h Interval in Dogs Decreases the Bacterial Contamination of Canine Ejaculates. Theriogenology.

[7] Agudelo-Yepes S., Puerta-Suárez J., Carrillo-Gonzalez D.F., Cardona Maya W.D. (2023). Bacteriospermia Assessment and Its Relationship with Conventional Seminal Parameters in Stud Dogs Ejaculates (Canis Familiaris). J. Hell. Vet. Med. Soc..

[8] Oguejiofor C. (2018). Sperm Defects and Infertility Caused by Bacterial Infection of the Reproductive Tract in an Adult Male Dog: A Case Report. Asian Pac. J. Reprod..

[9] Luciani M., Krasteva I., Di Febo T., Perletta F., D’Onofrio F., De Massis F., D’Alterio N., Sacchini F., Tittarelli M. (2023). Proteomics and Bioinformatics Investigations to Improve Serological Diagnosis of Canine Brucellosis. Proteom. Clin. Appl..

[10] Razin1 S., Tully J.G. (1970). Cholesterol Requirement of Mycoplasmas. J. Bacreriology.

[11] Schulz M., Sánchez R., Soto L., Risopatrón J., Villegas J. (2010). Effect of *Escherichia coli* and Its Soluble Factors on Mitochondrial Membrane Potential, Phosphatidylserine Translocation, Viability, and Motility of Human Spermatozoa. Fertil. Steril..

[12] Tamiozzo P.J. (2022). Mycoplasma Maculosum and Mycoplasma Spumans Associated with Fertility Disorders in Dogs from a Bernese Mountain Dog Kennel. Rev. Argent. Microbiol..

[13] Meyers-Wallen V.N. (2007). Unusual and Abnormal Canine Estrous Cycles. Theriogenology.

[14] Laber G., Holzmann A. (1977). Experimentally Induced Mycoplasmal Infection in the Genital Tract of the Male Dog. Theriogenology.

[15] Johnston S.D., Root Kustritz M.V., Olson P.N.S. (2001). Canine and Feline Theriogenology.

[16] Fowlkes D.M., Dooher G.B., O’leary W.M. (1975). Evidence by Scanning Electron Microscopy for an Association between Spermatozoa and T-Mycoplasmas in Men of Infertile Marriage Sc. Fertil Steril.

[17] Domrazek K., Jurka P. (2024). Prevalence of *Chlamydophila* Spp. and Canid Herpesvirus-1 in Polish Dogs. Vet. World.

[18] Martins M.I.M., Souza F.F.d., Oba E., Lopes M.D. (2006). The Effect of Season on Serum Testosterone Concentrations in Dogs. Theriogenology.

[19] Frank L.A., Mullins R., Rohrbach B.W. (2010). Variability of Estradiol Concentration in Normal Dogs. Vet. Dermatol..

[20] Shiel R.E., Sist M., Nachreiner R.E., Ehrlich C.P., Mooney C.T. (2010). Assessment of Criteria Used by Veterinary Practitioners to Diagnose Hypothyroidism in Sighthounds and Investigation of Serum Thyroid Hormone Concentrations in Healthy Salukis. J. Am. Vet. Med. Assoc..

[21] Domrazek K., Kaszak I., Kanafa S., Sacharczuk M., Jurka P. (2023). The Influence of *Mycoplasma* Species on Human and Canine Semen Quality: A Review. Asian J. Androl..

[22] Chalker V.J. (2005). Canine Mycoplasmas. Res. Vet. Sci..

[23] Chalker V.J., Brownlie J. (2004). Taxonomy of the Canine Mollicutes by 16S RRNA Gene and 16S/23S RRNA Intergenic Spacer Region Sequence Comparison. Int. J. Syst. Evol. Microbiol..

[24] Root Kustritz M.V. (2007). The Value of Canine Semen Evaluation for Practitioners. Theriogenology.

[25] Partyka A., Nizanski W., Ochota M. (2012). Methods of Assessment of Cryopreserved Semen. Current Frontiers in Cryobiology.

[26] Björndahl L., Söderlund I., Kvist U. (2003). Evaluation of the One-step Eosin-nigrosin Staining Technique for Human Sperm Vitality Assessment. Human Reprod..

[27] Takagi H. (2001). Interactive Evolutionary Computation: Fusion of the Capabilities of EC Optimization and Human Evaluation. Proc. IEEE.

[28] Altman D., Machin D., Bryant T., Gardner M. (2000). Statistics with Confidence: Confidence Intervals and Statistical Guidelines.

[29] Schäfer-Somi S., Spergser J., Aurich C. (2009). Bacteria and Mycoplasms in Canine Ejaculates a Retrospective Survey. Wien. Tierarztl. Monatsschr..

[30] Maksimović Z., Maksimović A., Halilbašić A., Rifatbegović M. (2018). Genital Mycoplasmas of Healthy Bitches. J. Vet. Diagn. Investig..

[31] Goericke-Pesch S., Weiss R., Wehrend A. (2011). Bacteriological Findings in Different Fractions of Canine Ejaculates Showing Normospermia, Teratozoospermia or Azoospermia. Aust. Vet. J..

[32] Manoni F., Gessoni G., Alessio M.G., Caleffi A., Saccani G., Silvestri M.G., Poz D., Ercolin M., Tinello A., Valverde S. (2011). Mid-Stream vs. First-Voided Urine Collection by Using Automated Analyzers for Particle Examination in Healthy Subjects: An Italian Multicenter Study. Clin. Chem. Lab. Med..

[33] Banchi P., Bertolotti L., Spanoghe L., Ali Hassan H., Lannoo J., Domain G., Henzel K.S., Gaillard V., Rota A., Van Soom A. (2024). Characterization of the Semen Microbiota of Healthy Stud Dogs Using 16S RNA Sequencing. Theriogenology.

[34] Johnston S.D. (1991). Performing a Complete Canine Semen Evaluation in a Small Animal Hospital. Vet. Clin. N. Am. Small Anim. Pract..

[35] Chen L., Deng H., Cui H., Fang J., Zuo Z., Deng J., Li Y., Wang X., Zhao L. (2018). Inflammatory Responses and Inflammation-Associated Diseases in Organs. Oncotarget.

[36] Root Kustritz M.V., Johnston S.D., Olson P.N., Lindeman C.J. (2005). Relationship between Inflammatory Cytology of Canine Seminal Fluid and Significant Aerobic Bacterial, Anaerobic Bacterial or Mycoplasma Cultures of Canine Seminal Fluid: 95 Cases (1987–2000). Theriogenology.

[37] Jagódka D., Kaczorek-Łukowska E., Graczyk R., Socha P. (2023). Vaginal Aerobic Bacteria of Healthy Bitches and Those with Fertility Problems. Pol. J. Vet. Sci..

[38] Zduńczyk S., Jurczak A. (2008). Incidence of *Mycoplasma canis* in the Vagina in Three Groups of Bitches. Bull. Vet. Inst. Pulawy.

[39] Pitorri F., Dell’Orco M., Carmichael N., Barker E.N., Hollywood M., Tasker S. (2012). Use of Real-time Quantitative PCR to Document Successful Treatment of *Mycoplasma haemocanis* Infection with Doxycycline in a Dog. Vet. Clin. Pathol..

[40] Bytesnikova Z., Richtera L., Smerkova K., Adam V. (2019). Graphene Oxide as a Tool for Antibiotic-Resistant Gene Removal: A Review. Environ. Sci. Pollut. Res..

[41] Patangia D.V., Anthony Ryan C., Dempsey E., Paul Ross R., Stanton C. (2022). Impact of Antibiotics on the Human Microbiome and Consequences for Host Health. Microbiologyopen.

[42] Melgarejo T., Oakley B.B., Krumbeck J.A., Tang S., Krantz A., Linde A. (2021). Assessment of Bacterial and Fungal Populations in Urine from Clinically Healthy Dogs Using Next-Generation Sequencing. J. Vet. Intern. Med..

