# Supplementary material for: The Impact of Microorganisms on Canine Semen Quality
Source: Animals (Basel). 2024 Apr 23;14(9):1267. doi: 10.3390/ani14091267 (PMC11083039; doi:10.3390/ani14091267)
Supplement: Supplementary file 1 [file animals-14-01267-s001.zip › animals-2973939-supplementary.pdf]

Table S1. Breeds of dogs of the study population

Breed	Number of dogs	Percentage
Crossbreed	3	4.8
Pedigree	60	95.2
Border Collie	6	10.0
Springer Spaniel	3	5.0
English Mastiff	3	5.0
Bernese Mountain Dog	2	3.3
Polish Lowland Sheepdog	2	3.3
Dandie Dimond Terrier	2	3.3
German Shepherd	2	3.3
Nova Scotia Duck Tolling Retriever	2	3.3
Lagotto Romagnolo	2	3.3
Whippet	2	3.3
Briard	2	3.3
Belgian Shepherd	2	3.3
Boston Terrier	2	3.3
Labrador Retriever	2	3.3
Sheltie	1	1.7
American Staffordshire Terrier	1	1.7
Papillon	1	1.7
Brussels Griffon	1	1.7
Samoyed	1	1.7
Appenzeller	1	1.7
Shiba Inu	1	1.7
White Swiss Shepherd	1	1.7
Pitbull	1	1.7
Small Münsterländer	1	1.7
Miniature Schnauzer	1	1.7
Miniature Dachshund	1	1.7
Thai Ridgeback	1	1.7
Boxer	1	1.7
Miniature Spitz	1	1.7
Dachshund	1	1.7
Pug	1	1.7
Flat coated retriever	1	1.7
Golden Retriever	1	1.7
German Shorthaired Pointer	1	1.7
Beagle	1	1.7
Polish Hound	1	1.7
Polish Tatra Sheepdog	1	1.7
Great Dane	1	1.7
Welsh Corgi Pembroke	1	1.7
Chinese Crested Dog	1	1.7

Table S2. Demographic and hormonal characteristics of dogs from *Mycoplasma*-positive and *Mycoplasma*-negative group

**Characteristics** [a]	***Mycoplasma*-positive (n = 38)**	***Mycoplasma*-negative (n = 25)**	**p-value**
**Demographic characteristics**			
Pedigree [b]	36 (94.7%)	24 (96.0%)	0.999
Age [years]	2.3, 1.5 – 4.0 (1.0 – 8.0)	3.5, 2.0 – 6.0 (1.0 – 8.0)	0.078
Body weight [kg]	22, 11 – 30 (3 – 120)	25, 16 – 34 (4.4 – 53)	0.779
**Hormones**			
Estradiol [pg/ml]	11.3, 5.0 – 17.8 (<9 – 47.2)	13.1, 5.0 – 16.0 (<9 – 40.0)	0.662
Testosterone [ng/ml]	4.0, 2.8 – 5.7 (1.0 – 12.0)	3.7, 1.6 – 5.6 (1.0 – 9.6)	0.357
Total thyroxin [nmol/l]	26.0, 18.6 – 30.7 (3.0 – 56.5)	25.0, 17.8 – 32.8 (11.0 – 40.0)	0.961

[a] presented as the median, interquartile range and range and compared using the Mann-Whitney U test unless otherwise stated

[b] presented as the count and percentage and compared using the Fisher exact test

Table S3. Influence of the presence of *Mycoplasma* in the semen on semen characteristics

**Semen characteristics**	***Mycoplasma*-positive (n = 38 or 37 [a])**		***Mycoplasma*-negative (n = 25)**		**p-value**
	Median	Interquartile range (Range)	Median	Interquartile range (Range)	
Semen volume [ml]	2.4	1.5 – 3.0 (0.6 – 5.0)	2.5	1.5 – 4.0 (0.4 – 5.0)	0.707
pH	6.0	6.0 – 6.5 (5.0 – 7.5)	6.0	6.0 – 6.5 (3.0 – 7.5)	0.671
Sperm concentration [×10^6/ml]	361.3	223.7 – 615.3 (42.2 – 1649.3)	370.2	185.7 – 513.2 (45.3 – 822.1)	0.358
Total sperm number [×10^6]	734.6	461.6 – 1260 (159.5 – 3298.7)	570.2	328.9 – 1401.7 (113.4 – 2093.6)	0.295
Azoospermia / Oligospermia [b]	4 / 38 (10.5%)		2 / 25 (8.0%)		0.999
Round cell count [×10^6/ml]	1.0	0.5 – 3.0 (0 – 9.8)	0.9	0.3 – 3.0 (0 – 17.7)	0.790
**Sperm morphology**					
Normal spermatozoa [%]	93.5	87.5 – 95.0 (58.5 – 98.5)	0.9	89.0 – 95.0 (62.0 – 99.0)	0.943
Head abnormalities [%]	1.5	1.0 – 3.0 (0 – 18.0)	2.0	1.0 – 4.0 (0 – 5.5)	0.994
Midpiece abnormalities [%]	2.0	1.5 – 3.5 (0 – 9.0)	2.0	1.5 – 3.5 (0 – 20)	0.943
Tail abnormalities [%]	2.0	1.0 – 6.0 (0 – 29.5)	3.0	1.0 – 4 .0 (0 – 13)	0.880
Abnormal spermatozoa [%]	6.5	5.0 – 12.5 (1.5 – 41.5)	7.5	5.0 – 11.0 (1.0 – 38)	0.943
**Sperm motility**					
Total motility [%]	93.3	88.0 – 96.5 (34.4 – 98.6)	93.8	87.7 – 95.6 (68.8 – 99.8)	0.977
Progressive motility [%]	28.8	20.4 – 39.0 (0.7 – 55.9)	35.0	25.1 – 40.9 (9.1 – 54.9)	0.162

Medium-progressive motility [%]	33.9	26.0 – 47.8 (4.6 – 71.2)	30.9	26.7 – 42.2 (8.9 – 78.7)	0.430
Non-progressive motility [%]	23.6	17.1 – 29.5 (10.3 – 49.5)	23.5	19.6 – 29.3 (5.1 – 38.6)	0.698
Spherical tracks [%]	43.8	31.8 – 55.4 (1.2 – 78.7)	36.2	30.1 – 46.5 (16.0 – 83.7)	0.251
Rapid motility [%]	58.5	44.7 – 70.2 (3.7 – 86.1)	55.1	48.3 – 67.0 (29.8 – 94.2)	0.954
Medium motility [%]	24.1	18.5 – 31.7 (7.2 – 48.6)	25.2	17.6 – 30.3 (5.1 – 53.4)	0.920
Slow motility [%]	7.0	3.9 – 10.9 (0.7 – 38.8)	6.9	5.0 – 10.7 (0.5 – 35.7)	0.977
Mucus penetration [%]	29.7	20.8 – 41.1 (2.4 – 64.9)	32.5	20.7 – 39.4 (4.2 – 50.5)	0.869
Viability [%]	92.0	88.0 – 94.0 (40.0 – 97.0)	91.0	87.0 – 95.0 (54.0 – 98.5)	0.863
**Bacterial coinfection**					
Bacteria in semen [b]	6 / 38 (15.8%)		2 / 25 (8.0%)		0.461

[a] sperm characteristics for 62 dogs which had sperms in semen

[b] presented as the count and percentage and compared using the Fisher exact test
